# Supplementary material for: Enumerating all possible biosynthetic pathways in metabolic networks
Source: Sci Rep. 2018 Jul 2;8:9932. doi: 10.1038/s41598-018-28007-7 (PMC6028704; doi:10.1038/s41598-018-28007-7)
Supplement: Supplementary file 1 — Supplementary Information [file 41598_2018_28007_MOESM1_ESM.pdf]

Enumerating all possible biosynthetic pathways in metabolic networks

Aarthi Ravikrishnan[1,2,3], Meghana Nasre[4,*], and Karthik Raman[1,2,3,*]

[1]Department of Biotechnology, Bhupat and Jyoti Mehta School of Biosciences, Chennai – 600036, INDIA
[2]Initiative for Biological Systems Engineering (IBSE)
[3]Robert Bosch Centre for Data Science and Artificial Intelligence (RBC-DSAI)

[4]Department of Computer Science and Engineering, Indian Institute of Technology (IIT) Madras, Chennai – 600036, INDIA
[*]meghana@iitm.ac.in, kraman@iitm.ac.in

1 SUPPLEMENTARY FIGURES

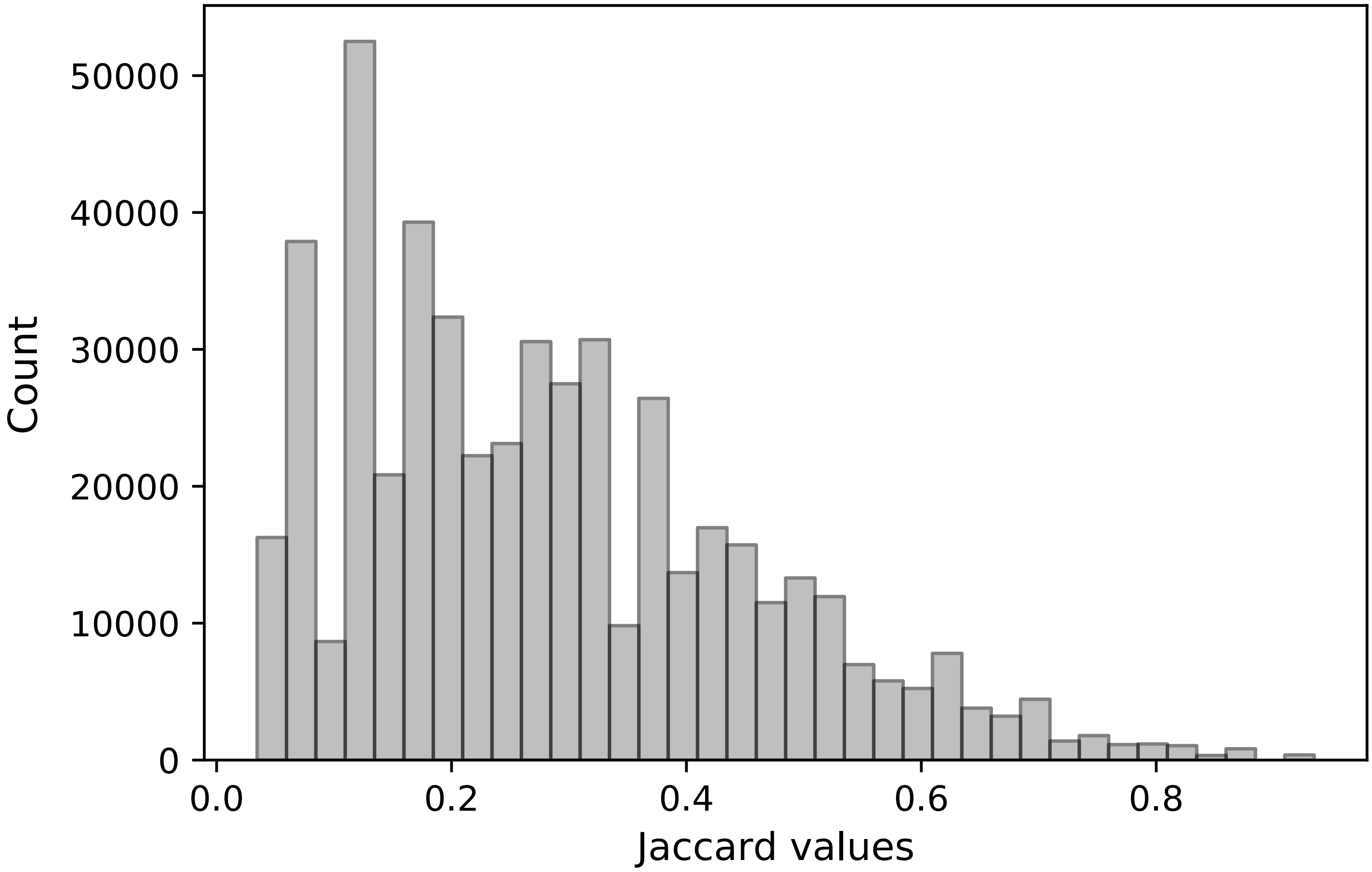

Figure S1: **Histogram showing the Jaccard values** of the glycolysis sub-networks generated by MetQuest. Note that very few viz., ≈ 202 pairs of sub-networks, have Jaccard values greater than 0.93.

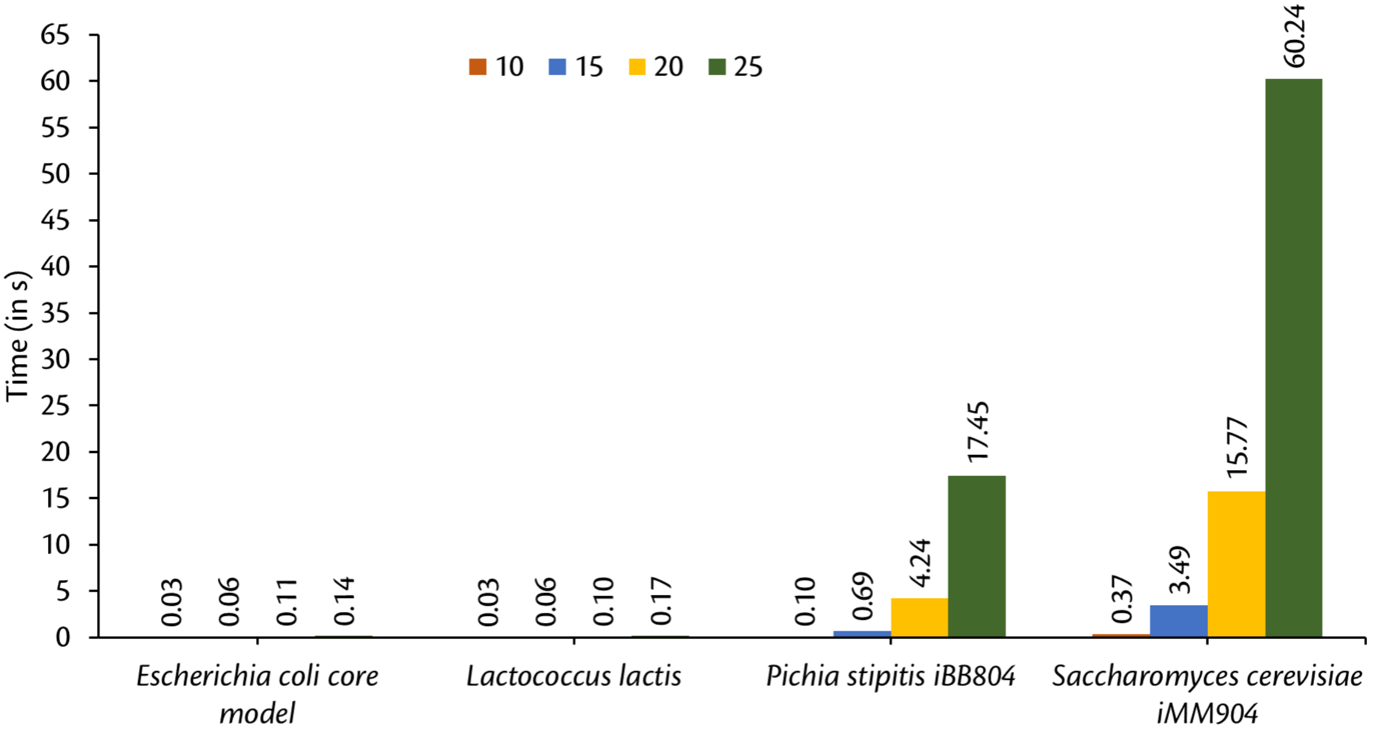

Figure S2: **Running times of MetQuest.** *X-axis* represents the bi-partite graphs $G(M, R, E)$, corresponding to the four genome-scale models in the order of increasing number of reactions, viz. *Escherichia coli* core model ($|M| = 72, |R| = 95, |E| = 566$), *Lactococcus lactis* ($|M| = 650, |R| = 754, |E| = 4207$), *Pichia stipitis i*BB804 ($|M| = 971, |R| = 1371, |E| = 7699$) and *Saccharomyces cerevisiae i*MM904 ($|M| = 1228, |R| = 1577, |E| = 8386$). The *Y-axis* shows the time taken to compute all the sub-networks of specified size cut-offs $\beta = 10, 15, 20, 25$, represented by four different coloured bars. As the model size and the cut-off β increase, the time taken to compute the sub-networks also increases. The seed metabolite set consists of metabolites listed in Supplementary Table S5, and the target metabolite was pyruvate.

2 SUPPLEMENTARY TABLES

Method	Current status†	Type	Ref.
PathFinder	Link appears broken	Visualisation of biochemical transformation	[1]
PathMiner	Link appears broken	Represent the compounds based on chemical descriptors and carry out an uninformed search	[2]
MetaPath	Link exists but no page to submit query	Calculates the *scope* of metabolic networks given a set of starting *seed*	[3]
NeAT	Link to find paths appears broken	Toolbox for analysis of biological networks	[4]
Rahnuma	Link appears broken	Hypergraph-based method that performs DFS on hypergraph to find routes	[5]
ReTrace (Python code)	—	Identifies branching metabolic pathways in atom-level representation of metabolic networks	[6]
FMM	Active	Constructs metabolic pathways between metabolites using substrate graph representation	[7]
PathPred	Active	Generates the pathways based on the structure transformation patterns and its comparison with reference pathway	[8]
BPAT-S/BPAT-M	Active	Identifies branched pathways by first determining linear pathways and combining them	[9]
MRSD	Link appears broken	Searches and designs routes based on the weighted compound transform digraph	[10]
Metabolic Tinker	Link appears broken, C# code available	Searches for thermodynamically feasible paths in metabolic universe using a tailored heuristic search strategy	[11]
RouteSearch	Active	Branch-and-Bound search and involves constructing a network of atom mappings to facilitate efficient searching	[12]
FindPath (MATLAB code)	—	Using chemical reaction database generates possible metabolic pathways and uses Genome-Scale Metabolic Model to find most efficient synthetic pathway	[13]
ATLAS	Active	Finds possible transformations between two metabolites using reactions from KEGG and other reactions specific to ATLAS	[14]
MRE	Active	Provides organism specific data from KEGG online tool for heterologous biosynthesis pathway design	[15]

†Accessed on October 26, 2017

Table S1: **Existing path-finding methods and algorithms.** Table shows some of the prominent path-finding algorithms and their descriptions. These algorithms/methods are based on different heuristics, and aim to infer/predict the routes of conversions from source to the target molecules. We note that many of these methods appear to have a broken link.

iJO1366 ACP_c: Acyl carrier protein
iJO1366 adp_c: ADP C10H12N5O10P2
iJO1366 amp_c: AMP C10H12N5O7P
iJO1366 atp_c: ATP C10H12N5O13P3
iJO1366 co2_c: CO2
iJO1366 coa_c: Coenzyme A
iJO1366 h2o_c: H2O
iJO1366 h2o_e: H2O
iJO1366 h2o_p: H2O
iJO1366 h_c: H+
iJO1366 nad_c: Nicotinamide adenine dinucleotide
iJO1366 nadh_c: Nicotinamide adenine dinucleotide - reduced
iJO1366 nadp_c: Nicotinamide adenine dinucleotide phosphate
iJO1366 nadph_c: Nicotinamide adenine dinucleotide phosphate - reduced
iJO1366 pi_c: Phosphate
iJO1366 pi_p: Phosphate
iJO1366 ppi_c: Diphosphate
glc__D_e: D-glucose

Table S2: Seed set of metabolites *S* for identifying pathways from glucose to pyruvate in *E. coli i*JO1366. While simulating, we add the model ID to the metabolite name, for example, atp_c in this simulation will be 'iJO1366 atp_c'.

iMM904 accoa_c: Acetyl-CoA
iMM904 accoa_m: Acetyl-CoA
iMM904 ACP_c: Acyl carrier protein
iMM904 ACP_m: Acyl carrier protein
iMM904 adp_c: ADP C10H12N5O10P2
iMM904 adp_m: ADP C10H12N5O10P2
iMM904 atp_c: ATP C10H12N5O13P3
iMM904 atp_m: ATP C10H12N5O13P3
iMM904 co2_c: CO2
iMM904 co2_m: CO2
iMM904 coa_c: Coenzyme A
iMM904 coa_m: Coenzyme A
iMM904 h2o_c: H2O
iMM904 h2o_m: H2O
iMM904 h_c: H+
iMM904 h_m: H+
iMM904 nad_c: Nicotinamide adenine dinucleotide
iMM904 nad_m: Nicotinamide adenine dinucleotide
iMM904 nadh_c: Nicotinamide adenine dinucleotide - reduced
iMM904 nadh_m: Nicotinamide adenine dinucleotide - reduced
iMM904 nadp_c: Nicotinamide adenine dinucleotide phosphate
iMM904 nadp_m: Nicotinamide adenine dinucleotide phosphate
iMM904 nadph_c: Nicotinamide adenine dinucleotide phosphate - reduced
iMM904 nadph_m: Nicotinamide adenine dinucleotide phosphate - reduced
iMM904 nh4_c: Ammonium
iMM904 pi_c: Phosphate
iMM904 pi_m: Phosphate
iMM904 ppi_c: Diphosphate
iMM904 ppi_m: Diphosphate
iMM904 glc_DASH_D_c: D-glucose

Table S3: Seed set of metabolites *S* for identifying pathways from D-glucose to L-phenylalanine of length 28 in *S. cerevisiae i*MM904.

catechol_e: Catechol

iJN746 adp_c:	ADP C10H12N5O10P2
iJN746 adn_c:	Adenosine
iJN746 amp_c:	AMP C10H12N5O7P
iJN746 atp_c:	ATP C10H12N5O13P3
iJN746 co2_c:	CO2 CO2
iJN746 coa_c:	CO2 CO2
iJN746 h2o_c:	H2O H2O
iJN746 h2o_p:	H2O H2O
iJN746 h_c:	H+
iJN746 h_p:	H+
iJN746 nad_c:	Nicotinamide adenine dinucleotide
iJN746 nadh_c:	Nicotinamide adenine dinucleotide - reduced
iJN746 nadp_c:	Nicotinamide adenine dinucleotide phosphate
iJN746 nadph_c:	Nicotinamide adenine dinucleotide phosphate - reduced
iJN746 pi_c:	Phosphate
iJN746 pi_p:	Phosphate
iJN746 ppi_c:	Diphosphate
iJN746 o2_e:	O2 O2
iJN746 co2_e:	CO2 CO2
iJN746 succoa_c:	Succinyl-CoA

Table S4: Seed set of metabolites S for identifying pathways from catechol to fumarate in *P. putida* *i*JN746.

ACP_c:	Acyl carrier protein
adp_c:	ADP C10H12N5O10P2
amp_c:	AMP C10H12N5O7P
atp_c:	ATP C10H12N5O13P3
co2_c:	CO2
coa_c:	Coenzyme A
h2o_c:	H2O
h2o_p:	H2O
h_c:	H+
h_e:	H+
nad_c:	Nicotinamide adenine dinucleotide
nadh_c:	Nicotinamide adenine dinucleotide - reduced
nadp_c:	Nicotinamide adenine dinucleotide phosphate
nadph_c:	Nicotinamide adenine dinucleotide phosphate - reduced
pi_c:	Phosphate
pi_p:	Phosphate
ppi_c:	Diphosphate
glc_DASH_D_e:	D-glucose

Table S5: This set represents the common pool of seed metabolites used in all the simulations. As stated earlier, the metabolites were renamed based on the models where the pathways were identified. Note that following metabolites were found in the graph, and hence were removed Ecoli_core_model pi_p, Ecoli_core_model ppi_c, Ecoli_core_model h2o_p, Lactococcus_lactis_MG1363 pi_p, Lactococcus_lactis_MG1363 h2o_p, P1 pi_p and P1 h2o_p

3 SUPPLEMENTARY ALGORITHM

Algorithm S1 Guided BFS – An algorithm to to identify the scope of metabolites

Input: $G(M, R, E)$, where M is the set of metabolites in the metabolic network, R is the set of reactions and E is the set of edges, Seed metabolites S (containing the source metabolite(s)) and a set of target nodes T.
Output: Scope M_s of the seed metabolite set S.
$R_s \leftarrow succ(m'_s), \forall m'_s \in S$ ▷ R_s is the set of source reaction nodes
$R_t \leftarrow pred(t), \forall m_t \in T$ ▷ R_t is the set of target reaction nodes
$Q = \varnothing$ ▷ Queue containing reactions whose predecessors are present in S
$R_{start} = \varnothing$
for each $r \in R_s$ **do**
    if $pred(r) \subseteq S$ **then**
        $visited(r)$ = true
        $M_{succ} \leftarrow succ(r)$ ▷ M_{succ} is a set of metabolite nodes and $M_{succ} \subseteq M$
        $S = S \cup M_{succ}$
        $R_{start} = R_{start} \cup r$
for each $r \in R_{start}$ **do**
    for each $m_s \in succ(r)$ **do**
        for every $r' \in succ(m_s)$ **do**
            if metabolites to trigger reaction r' are present in S & visited $(r') \neq$ true **then**
                Enqueue r' to Q
while $Q \neq \varnothing$ **do**
    $r =$ dequeue(Q)
    $visited(r)$ = true
    $M_{succ} \leftarrow succ(r)$ ▷ M_{succ} is a set of metabolite nodes and $M_{succ} \subseteq M$
    $S = S \cup M_{succ}$
    for each $m_s \in M_{succ}$ **do**
        for every $r' \in succ(m_s)$ **do**
            if metabolites to trigger reaction r' are present in S & visited $(r) \neq$ true **then**
                Enqueue r' to Q

Note: Here *succ* refers to the successors, i.e., the metabolites produced by a reaction and *pred* refers to the predecessors, i.e., the precursor metabolites required by every reaction.

4 SUPPLEMENTARY METHODS

4.1 Optimisations carried out

In this section, we discuss the different optimisations carried out to improve the running times of the algorithm. Broadly, these optimizations pertain to the reduction in the number of partitions generated and pruning the number of reactions assessed at every stage of iteration.

1. While generating the partition of numbers, we observe that many partitions get repeated for generating the same value over successive iterations. For instance, if we should generate a sum of 10, while iterating over $k = 8$ and 9, using two numbers which can take values between $(3 \text{ } to \text{ } 10)$ and $(2 \text{ } to \text{ } 10)$ respectively, we would be generating the following partitions:

    - $k = 8$ partitions include $(3, 7), (4, 6), (5, 5), (7, 3)$
    - $k = 9$ partitions include $(3, 7), (4, 6), (5, 5), (7, 3), (8, 2)$

    Although many partitions overlap, few new partitions are generated with increasing j values. To generate only the new partitions, we break the procedure into two different stages.

2. In the first round of calculations, we define a new variable t whose value is given by

$$t = \left\lfloor \frac{\ell}{k-1} \right\rfloor$$

where l is the sum that we intend to generate. Depending on the value of t, we assign the value of $k-1$ to t inputs (in multiple combinations) and calculate the values that $n-t$ variables can take up to generate the given sum. Based on these values, we carry out Step 5 of Algorithm 1 and fill up the corresponding entry in the table. In this case, $\ell = 10$, $(k-1) = 8$, thus $t = 1$. Now, we assign the value of 8 to 1 input and calculate the value of other input such that both sum to 10. We do this $\binom{n}{t}$ times to assess all combinations.

3. In the second round, we generate partition for all the values which have not been generated so far. For instance, when $k = 8$ and $k = 9$, we would generate a partition of numbers that sum (ℓ) between 7 to 14 and 8 to 16 respectively. Thus, once we intend to evaluate $k = 9$, we generate a full set of partitions only for the sum (ℓ) 15 and 16. In the second round of calculations, we repeat Step 4 and Step 5, only for the sum that has not been generated in the previous step. For the case shown above, we would be performing this step when $k = 9, \ell = 15, \ell = 16$, since partitions for these sums have not been evaluated so far.

4. We prune the number of reactions by taking into consideration only those reaction nodes can be *visited* (based on the availability of precursors) while carrying out Guided BFS over original and reversed graph. To obtain a reversed graph, we merely reverse the directions of the reactions in the original graph. Scope M_s, obtained at the end of traversal on the original graph, is fed as an input to the reversed graph. The traversal starts from the target and stops when the source is reached. This step eliminates reactions that are not a part of the pathway from source to target.

5. To generate the sum ℓ, we use values between ℓ_m and $k-1$, and not 0 to $k-1$, since ℓ_m already has the information about when the metabolite can be first visited. Thus, computation of partitions using numbers between 0 and ℓ_m, would not be useful.

4.2 Generation of partitions (generatePartitions function)

generatePartitions: This function takes as input 3 values ℓ, s, j. Here ℓ is the sum that we want to generate using s non-negative integers which are upper-bounded by j. The function returns all possible ways of generating the sum ℓ. For example, when **generatePartitions**(5, 3, 3) is called the function will return:

- $\langle 3,2,0\rangle \langle 3,1,1\rangle \langle 3,0,2\rangle$
- $\langle 2,3,0\rangle \langle 2,2,1\rangle \langle 2,1,2\rangle \langle 2,0,3\rangle$
- $\langle 1,3,1\rangle \langle 1,2,2\rangle \langle 1,1,3\rangle$
- $\langle 0,3,2\rangle \langle 0,2,3\rangle$

Note that the partitions of the type $\langle 4,1,0\rangle$ are not generated since $4 > 3$.

4.3 Computation of partitions till $n \times (k-1)$

In this section, we explain why we should generate partitions till $n \times (k-1)$ by illustrating it on a small toy-network. Consider a directed bipartite graph as shown in Figure S3. If we were to traverse from source M_1 to target M_5, with A_1 as the seed metabolite set S, and cut-off j 3 and k 100, following would be the steps followed by MetQuest:

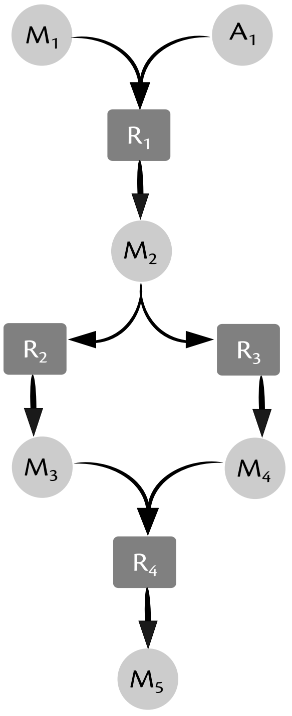

Cutoff	Partitions
2	$(1,1),(2,0),(0,2)$
3	$(0,3),(1,2),(2,1),(3,0)$
4	$(0,4),(1,3),(2,2),(3,1),(4,0)$

	0	1	2	3	4
M_1	$\emptyset$	$\perp$	$\perp$	$\perp$	$\perp$
A_1	$\emptyset$	$\perp$	$\perp$	$\perp$	$\perp$
M_2	$\perp$	$\{R_1\}$	$\perp$	$\perp$	$\perp$
M_3	$\perp$	$\perp$	$\{R_1, R_2\}$	$\perp$	$\perp$
M_4	$\perp$	$\perp$	$\{R_1, R_3\}$	$\perp$	$\perp$
M_5	$\perp$	$\perp$	$\perp$	$\perp$	$\{R_1, R_2, R_3, R_4\}$

Figure S3: **Top panel :** Toy reaction network with 4 reactions (Dark gray rectangles) and 6 metabolites (Light gray circles). **Centre panel :** Table showing the partitions generated for various cutoffs for a reaction with number of inputs $n = 2$. **Bottom panel :** Output Table generated by MetQuest.

1. From Guided BFS, we can find M_s to be contain M_2, M_3, M_3 and M_5.

2. We generate the table of size (6,4), and fill the values against seed metabolites and the source as $\emptyset$. In every iteration, for every reaction $r \in R_v$, we check if the input reactants can be generated in $k-1$ reactions

3. In the first iteration $k = 1$, for every reaction $r \in R_v$, we check if the input reactants can be generated in $k-1$ to $n \times (k-1)$ reactions, i.e., in this case, it is 0 reactions. Based on the number of inputs r to the reaction, we partition $k-1$ using r integers, such that together they add up to $k-1$. While performing this over all the reactions, we find only reaction R_1, whose inputs satisfy the partitions. Thus, we fill the values $Table(M_2, 1) = R_1$, corresponding to metabolite M_2 generated by R_1. The other metabolites in the *Table* are filled as $\perp$, indicating that they cannot be produced in one reaction.

4. Similarly, in the second iteration, we consider all the reactions $r \in R_v$, and check if the input reactants can be generated in $k-1$ reactions to $n \times (k-1)$, i.e., 1 reaction. We find that only for the reactions R_2 and R_3, the inputs M_2 satisfy the partition. Thus, we fill in $Table(M_3, 2) = R_2 \cup Table(M_2, 1)$ and $Table(M_4, 2) = R_3 \cup Table(M_2, 1)$. The other metabolites in the table are filled as $\perp$.

5. Again in the third step, we consider all the reactions $r \in R_v$, and check if the input reactants can be generated in $k-1$ to $n \times (k-1)$ reactions, i.e., with 2 $(3-1)$ to 6 $(2 \times (3-1))$ reactions. We generate partitions of numbers for every reaction $r \in R_v$ and find only the inputs to reaction R_4 satisfy the partition generated i.e., (2,2) (Figure S3). Using these partitions, we fetch the corresponding entry from the table, i.e., $Table(M_3, 2)$ and $Table(M_4, 2)$. The entry $Table(M_3, 2) \cup Table(M_4, 2) \cup R_4$ would give R_1, R_2, R_3, R_4 which is filled in $Table(M_5, 4)$.

6. If we had restricted the generation of partitions till $(k-1)$, we would have missed this sub-network, which is derived using the partition of numbers generating a sum of 4 $(n \times (k-1))$. The final output table can be found in Figure S3.

4.4 Demonstration of MetQuest on a toy-network

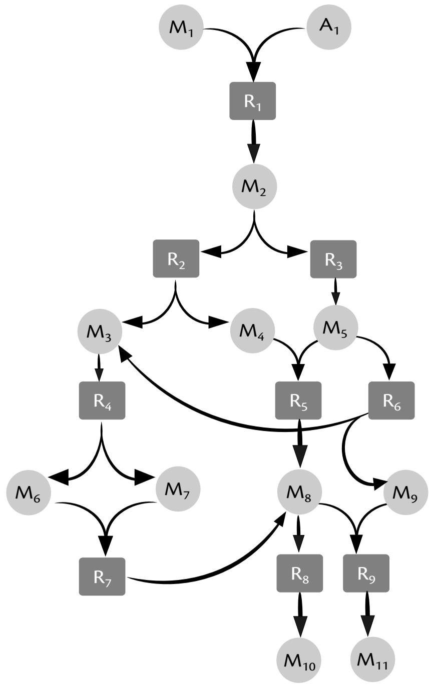

Figure S4: Toy reaction network with 9 reactions (Dark gray rectangles) and 11 metabolites (Light gray circles). This network is used to demonstrate how MetQuest works on a toy-network. The final output of pathways producing the metabolites can be found in Table S6. Note that M_1 and A_1 are the seed metabolites. We intend to find pathways of size $\beta = 7$ to target M_{11}.

The algorithm starts with the seed metabolite set S, which contains M_1 and A_1. From the first phase, we obtain the scope M_s of S. We then initialise the Table of size $|M_s| \times (\beta + 1)$. In the first stage, we fill in column 0 with $\emptyset$ against the entries corresponding to seed metabolites. We also check the reaction where these seed metabolites, i.e., M_1 and A_1, participate and determine the metabolite produced. We then fill the $Table(M_2, 1)$ as R_1, since this reaction involves the seed metabolites and produces M_2. From the next step, we start generating partition of numbers for every reaction, and fetch the corresponding values from the Table if they are present in the system. For instance, consider reaction R_2, which requires one metabolite M_2. Since we currently intend to fill column 2, we generate partitions that sum to 1 using 1 input. We then check if there are any entries against $Table(M_2, 1)$. We then perform a union of this reaction R_1 with the current reaction R_2 and fill it against $Table(M_3, 2)$. If there are multiple entries, we consider single entries at a time and perform a cross-product on the union of reaction sets. Note that the final Table size may be greater than β, since we compute partitions of numbers till $n \times (k-1)$, where $k = 1, \ldots, \beta$.

	0	1	2	3	4	5	6	7
A_1	$\emptyset$	$\perp$	$\perp$	$\perp$	$\perp$	$\perp$	$\perp$	$\perp$
M_1	$\emptyset$	$\perp$	$\perp$	$\perp$	$\perp$	$\perp$	$\perp$	$\perp$
M_2	$\perp$	$\{R_1\}$	$\perp$	$\perp$	$\perp$	$\perp$	$\perp$	$\perp$
M_3	$\perp$	$\perp$	$\{R_1, R_2\}$	$\{R_1 ,R_3, R_6\}$	$\perp$	$\perp$	$\perp$	$\perp$
M_4	$\perp$	$\perp$	$\{R_1, R_2\}$	$\perp$	$\perp$	$\perp$	$\perp$	$\perp$
M_5	$\perp$	$\perp$	$\{R_1, R_3\}$	$\perp$	$\perp$	$\perp$	$\perp$	$\perp$
M_6	$\perp$	$\perp$	$\perp$	$\{R_1, R_3, R_4\}$	$\{R_1, R_3, R_6, R_4\}$	$\perp$	$\perp$	$\perp$
M_7	$\perp$	$\perp$	$\perp$	$\{R_1, R_3, R_4\}$	$\{R_1, R_3, R_6, R_4\}$	$\perp$	$\perp$	$\perp$
M_8	$\perp$	$\perp$	$\perp$	$\perp$	$\{R_1, R_2, R_4, R_7\}$ $\{R_1,R_2, R_3, R_5\}$	$\{R_1, R_3, R_6,R_4, R_7\}$	$\perp$	$\perp$
M_9	$\perp$	$\perp$	$\perp$	$\{R_1, R_3, R_6\}$	$\perp$	$\perp$	$\perp$	$\perp$
M_{10}	$\perp$	$\perp$	$\perp$	$\perp$	$\perp$	$\{R_1, R_2, R_3, R_5, R_8\}$	$\{R_1, R_3, R_6, R_4, R_7, R_8\}$	$\perp$
M_{11}	$\perp$	$\perp$	$\perp$	$\perp$	$\perp$	$\perp$	$\{R_1, R_2, R_3, R_5, R_6, R_9\}$	$\{R_1, R_2, R_3, R_4, R_6, R_7, R_9\}$

Table S6: Table of sub-networks generated by the algorithm for the toy-network shown in Figure S4.

4.5 Dealing with cycles

Besides the linear and branched pathways, MetQuest, due to the nature of formulation, also effectively deals with cycles. As an example, consider a directed cyclic bipartite graph as shown in Figure S5. We apply MetQuest for a cut-off of five, with M_1,A_1 and M_4 as the source, seed and target metabolite respectively. In the first step of Guided BFS, all the reactions in the network are marked visited, and the scope of M_1 and A_1 are calculated. In the second step, while generating sub-networks, the table entries are sequentially filled. M_2 is generated by R_4 as a part of the cycle. While filling the entry corresponding to $Table(M_2, 4)$ using reaction R_4, we generate partitions using one number whose sum is three (since there is one input to reaction R_4). Using the output generated from previous iteration, $Table(M_4, 3) = R_1, R_2, R_3$. Since M_4 can be generated using 3 reactions, $Table(M_2, 4)$ will now be R_1, R_2, R_3, R_4. In the next iteration $k = 5$ and $R = R_2$, we generate partitions using one number to generate a sum of 4, and fetch the entry corresponding to $Table(M_2, 4)$. When we perform a cross-product of the values from $Table(M_2, 4)$ and the current reaction, we find that the number of unique reactions is 4 i.e, R_1, R_2, R_3 and R_4. Hence this entry cannot be filled against $Table(M_2, 5)$ and thus the iteration stops. Thus, algorithm converges after finding the first cycle, since the number of unique reactions in the path are same and no new partitions are generated.

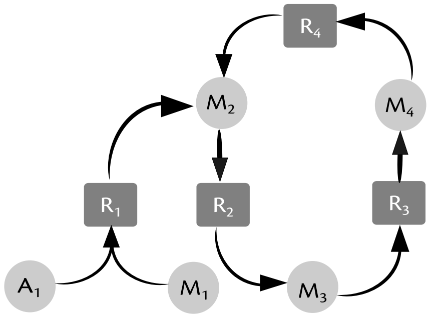

Figure S5: Cyclic pathway consisting 4 reactions and 5 metabolites. Light gray circles represent metabolites, dark gray rectangles represent reactions.

4.6 Implementation and detailed work-flow

We implemented MetQuest on Python 3.6, and is freely available at https://github.com/RamanLab/metquest. MetQuest is also available as a Python package from PyPI. Detailed documentation can be found at http://metquestdoc.readthedocs.io/. We read the SBML files using the functions in COBRAPy [16] and construct the bipartite graph using NetworkX package [17]. Note that in our implementation, while finding the branched networks, we restrict the combined number of the total sub-networks for each metabolite to ns_{total} (Consider a reaction $R1 : A + B \rightarrow C + D$, if there are 50 ways to synthesise A and 100 ways to synthesise B, there are 5000 ways to synthesize C and D. If $ns_{total} = 1000$, we consider the first 1000 networks only, although this can be changed. Thus, at the end of iterations, we get at least ns_{total} sub-networks.)

Further, the implementation of MetQuest also allows the user to seek answers to several interesting questions, such as

1. Can the target metabolite be produced using a pathway with n ($\leq \beta$) steps?
2. Can the metabolite be produced using (non-)cyclic pathways?
3. What are the reactions that are most important (i.e., frequent) for producing the target metabolite?
4. What are the most different pathways that produce the target metabolite?
5. What are the most frequently occurring exchanged metabolites between the organisms for producing the target of interest?

```
>>> import metquest
>>> metquest.example.run_this_example()
Filenames ['iJO1366.xml']
Number of edges in graph 12974
Number of nodes in graph 5659
Time taken 22.989315277667806

---------------
Summary
---------------
Number of metabolites in scope :  885
Target metabolite :  iJO1366 pyr_c
Pathway size cutoff :  15
Number of all branched pathways found from seed : 4787
Number of all branched pathways from seed whose size <= 15 : 1776
Minimum number of steps to produce  iJO1366 pyr_c  :  8
Number of branched pathways from source whose size <= 15 : 1007
iJO1366 pyr_c can be produced using cyclic pathway
Number of cyclic pathways whose size <= 15 : 6


One of the combination of most different pathways producing target metabolite
Note - There can be other combinations that can be found
For finding all the combinations, please use the function find_jaccard_between_paths
Pathway 1
glc__D_e glc__D_e --> iJO1366 glc__D_e
AMPN iJO1366 amp_c + iJO1366 h2o_c --> iJO1366 ade_c + iJO1366 r5p_c
DHAPT iJO1366 dha_c + iJO1366 pep_c --> iJO1366 dhap_c + iJO1366 pyr_c
GLCabcpp iJO1366 atp_c + iJO1366 h2o_c + iJO1366 glc__D_p --> iJO1366 adp_c + iJO1366 glc__D_c + iJO1366 h_c
GLCtex_copy2 iJO1366 glc__D_e --> iJO1366 glc__D_p
HEX1 iJO1366 atp_c + iJO1366 glc__D_c --> iJO1366 adp_c + iJO1366 g6p_c + iJO1366 h_c
ENO iJO1366 2pg_c --> iJO1366 h2o_c + iJO1366 pep_c
F6PA iJO1366 f6p_c --> iJO1366 dha_c + iJO1366 g3p_c
GAPD iJO1366 g3p_c + iJO1366 nad_c + iJO1366 pi_c --> iJO1366 13dpg_c + iJO1366 h_c + iJO1366 nadh_c
PGI iJO1366 g6p_c --> iJO1366 f6p_c
RPE iJO1366 ru5p__D_c --> iJO1366 xu5p__D_c
RPI iJO1366 r5p_c --> iJO1366 ru5p__D_c
TKT1 iJO1366 r5p_c + iJO1366 xu5p__D_c --> iJO1366 g3p_c + iJO1366 s7p_c
PGK iJO1366 13dpg_c + iJO1366 adp_c --> iJO1366 3pg_c + iJO1366 atp_c
PGM iJO1366 3pg_c --> iJO1366 2pg_c


Pathway 2
glc__D_e glc__D_e --> iJO1366 glc__D_e
MGSA iJO1366 dhap_c --> iJO1366 mthgxl_c + iJO1366 pi_c
NTD11 iJO1366 h2o_c + iJO1366 imp_c --> iJO1366 ins_c + iJO1366 pi_c
NTPP9 iJO1366 h2o_c + iJO1366 itp_c --> iJO1366 h_c + iJO1366 imp_c + iJO1366 ppi_c
PFK iJO1366 atp_c + iJO1366 f6p_c --> iJO1366 adp_c + iJO1366 fdp_c + iJO1366 h_c
ATPHs iJO1366 atp_c + iJO1366 h_c + iJO1366 h2o_c --> iJO1366 itp_c + iJO1366 nh4_c
GLCt2pp iJO1366 glc__D_p + iJO1366 h_p --> iJO1366 glc__D_c + iJO1366 h_c
GLYOX3 iJO1366 h2o_c + iJO1366 mthgxl_c --> iJO1366 h_c + iJO1366 lac__D_c
HEX7 iJO1366 atp_c + iJO1366 fru_c --> iJO1366 adp_c + iJO1366 f6p_c + iJO1366 h_c
FBA iJO1366 fdp_c --> iJO1366 dhap_c + iJO1366 g3p_c
GLCtex_copy1 iJO1366 glc__D_e --> iJO1366 glc__D_p
LDH_D iJO1366 lac__D_c + iJO1366 nad_c --> iJO1366 h_c + iJO1366 nadh_c + iJO1366 pyr_c
XYLI2 iJO1366 glc__D_c --> iJO1366 fru_c
INSt2pp_copy2 iJO1366 h_c + iJO1366 ins_c --> iJO1366 h_p + iJO1366 ins_p
```

```
TPI iJO1366 g3p_c --> iJO1366 dhap_c


Important reactions based on the frequency of occurrence are
ENO
GAPD
PGK
PGM
RPE
RPI
TKT1
```

4.6.1 Modules in MetQuest

The implementation of MetQuest has the following modules:

1. **construct_graph:** This module is used to construct the bipartite graph object using the metabolic models provided by the user.

2. **execute_metquest:** This module contains all the functions required to run MetQuest and perform the necessary calculations to infer the results. The functions include:

   (a) **find_important_reactions:** This function determines the most important reactions that are required to produce the target metabolite. These reactions are determined by enumerating the number of times a reaction occurs in the pathways producing the target metabolite from the source. The top 5 reactions, based on the frequency of occurrence are printed as the final result.

   (b) **find_jaccard_between_paths:** This function calculates the Jaccard index using the pathways producing the target metabolite from the source. Jaccard index $J(A, B)$ is defined as:

   $$J(A, B) = \frac{|A \cap B|}{|A \cup B|}$$

   where A and B are two different pathways (sets of reactions) producing the target metabolite.

   (c) **find_pathways_involving_exchange_mets:** This function identifies the pathways producing the target metabolites, which involve exchange metabolites. This function prints output only when a community of organisms is considered, i.e., when more than one metabolic network is used.

   (d) **find_pathways_starting_from_source:** This function finds all the pathways from the source metabolite, excluding the seed metabolites.

   (e) **print_summary:** This function prints the results summary obtained from the pathways, i.e.,

      i. Number of metabolites in scope
      ii. Target metabolite
      iii. Pathway size cutoff β
      iv. Number of all branched pathways found from seed
      v. Number of all branched pathways from seed whose size $\leq$ Pathway size cutoff β
      vi. Minimum number of steps to produce target metabolite
      vii. Number of branched pathways from source whose size $\leq$ Pathway size cutoff
      viii. If the target metabolite can be produced using cyclic pathway
      ix. Number of cyclic pathways whose size $\leq$ Pathway size cutoff β
      x. One of the combination of most different pathways producing target metabolite
      xi. Important reactions based on the frequency of occurrences

   (f) **write_output_to_file:** This function writes the pathways of sizes less than or equal to the cutoff from source to the target and seed metabolites to target. This function also writes cyclic pathways of sizes less than or equal to cut off from the source to target.

3. **fetch_reactions:** This module gets the data about the reactions and the metabolites from the models of multiple organisms. The functions in this module require as input the pathname where the .xml files are located. From this path, this function reads all the files using the functions in the COBRA toolbox and generates the stoichiometric model for these SBML models.

4. **generate_partitions:** This module generates combinations of numbers, which together add up to the desired sum.

5. **get_reactions_types:** This module determines the type of reactions, namely, reversible, irreversible and exchange reactions from the genome-scale metabolic model.

6. **guided_bfs:** This module carries out the *guided* breadth-first search (Phase 1), as explained in the main manuscript.

7. **pathway_assembler:** This module contains functions that carry out all the steps of Phase 2 of the algorithm, as explained in the main manuscript.

4.7 Formal proof of the algorithm

In this section, we present the correctness of our algorithm. The Phase 1 of the algorithm computes the scope M_s of the seed set S and for every metabolite m the value ℓ_m and for each reaction r the value ℓ_r. This phase is simply a modification of the standard breadth-first search algorithm, and we assume that M_s and ℓ_m and $\ell_r$are correctly generated. We now argue that the Algorithm MetQuest computes *Table* correctly. In particular, we prove the following:

- **Claim 1:** There exists a pathway $\mathcal{RS}$ of size k in the metabolic network that produces a metabolite m starting with the seed set S *if and only if* $Table[m][k]$ contains the pathway $\mathcal{RS}$.
- **Claim 2:** The entry $Table[m][k] = \perp$ *if and only if* there does not exist **any** pathway of size k in the metabolic network that produces m starting at the seed set S.

Proof: We prove the above statements by induction on the column index k. We begin by noting that for $k = 0$, the entry of the form $Table[m][0]$ for any metabolite m is correct. This is because our algorithm sets $Table[m][0] = \emptyset$ for only the seed metabolites S. For every other metabolite $Table[m][0]$ is set to $\perp$ indicating that m cannot be generated using zero reactions. By induction hypothesis assume that for all values of $k = 1, \ldots, \mathsf{col} - 1$, the above two claims are true. We now prove that for $k = \mathsf{col}$ both the statements hold. We split the proof into two parts.

- Assume that a pathway $\mathcal{RS}$ is generated by our algorithm when the for loop in Line 9 has $k = \mathsf{col}$ and the reaction in the for loop in Line 10 is r. We argue that such a pathway is a valid pathway in the metabolic network. For $k = \mathsf{col}$, Algorithm 1 considers all sum-values ℓ ranging from $\mathsf{col} - 1$ all the way up to $n \times (\mathsf{col} - 1)$, where n is the number of inputs to reaction r. Assume that $m_1, m_2, \ldots, m_n$ are the inputs to r. A particular sum-value ℓ is partitioned as $(p_1, p_2, \ldots, p_n)$ (using a call to generatePartitions). Note that this partition allows us to generate a pathway containing r which has a sub-pathway of size p_i for each m_i. Our algorithm then uses for $i = 1, \ldots, n$, $Table[m_i][p_i]$ to generate the pathway $\mathcal{RS}$. Finally, the algorithm adds it to the appropriate cell in the *Table* (using a call to populateTable).

  We note a subtle but important point here – our algorithm considers sum-values ℓ which are larger than $\mathsf{col} - 1$. However, to generate each partition, the maximum value that any p_i can take is at most $\mathsf{col} - 1$ (see Line 19 and Line 21 in Algorithm 1). This ensures that for a particular partition $(p_1, p_2, \ldots, p_n)$ for a sum value ℓ, when we query the entry $Table[m_i][p_i]$, we use entries of *Table* in columns indexed up to $\mathsf{col} - 1$. By induction hypothesis, all entries of *Table* in the columns up to $\mathsf{col} - 1$ are already computed correctly. Thus, using sub-pathways of size $p_1, p_2, \ldots, p_n$ (if they exist) and combining such sub-pathways with r we generate a pathway RS (Line 7 Algorithm 2) which must belong to the metabolic network.

  We also remark that since our algorithm considers sum-values all the way up to $n \times \mathsf{col} - 1$, the size of the pathways generated by our algorithm can be larger than col. Thus when the value of $k = \mathsf{col}$ our algorithm may add pathways to the entries in $Table[m][k']$ where $k' > \mathsf{col}$. We finally remark on the Step 3–4 of Algorithm 2 (populateTable). Note that for a particular partition $(p_1, p_2, \ldots, p_n)$ and the reaction r, if the entry $Table[m_i][p_i]$ is $\perp$ for some i, then it is clear that there is no way to generate a pathway which has a sub-network (pathway) of size p_i for the metabolite m_i. Thus our algorithm discards such a partition and proceeds with the next partition.

- Now consider any pathway $\mathcal{RS}$ of size k containing a particular reaction r in the metabolic network. We argue that at the end of the k-th iteration of the for loop in Line 9 of Algorithm 1, the pathway is generated by our algorithm. Let r be an n-input reaction with $m_1, m_2, \ldots, m_n$ as its inputs. Any pathway $\mathcal{RS}$ of size k containing

r must have sub-pathways $\mathcal{RS}_i$ for each i, where the sub-pathway $\mathcal{RS}_i$ produces m_i and size of $\mathcal{RS}_i$ is p_i. Here $0 \leq p_i \leq k-1$. Thus there exists a partition $(p_1, p_2, \ldots, p_n)$ denoting the size of each sub-pathway in $\mathcal{RS}$. We now note the important point. The sum-value $\ell = \sum_{i=1}^{n} p_i$ can range from $k-1$ to $n \times (k-1)$. The sum can be $k-1$ when each of the sub-pathway has distinct p_i many reactions. On the other hand, the sum can be $n \times (k-1)$ when there is a *single* sub-pathway of size $k-1$ producing *all* the n inputs to r. This is precisely the reason our algorithm considers all sum values ranging from $k-1$ to $n \times (k-1)$ (Line 13 in Algorithm 1). This step in the algorithm ensures that any partition that can possibly generate the pathway $\mathcal{RS}$ is considered by our algorithm.

5 SUPPLEMENTARY RESULTS

5.1 Pathways from D-glucose to L-phenylalanine of length 28 in *S. cerevisiae i*MM904

In this section, we enlist the two pathways of length 28 found by MetQuest in *S. cerevisiae i*MM904. The reactions (in blue colour) represent the target reactions producing L-phenylalanine.

Pathway 1

**PSCVTi:**	pep_c + skm5p_c → pi_c + 3psme_c
**ENO:**	2pg_c → pep_c + h2o_c
**GLUDyi:**	akg_c + h_c + nadph_c + nh4_c → nadp_c + h2o_c + glu_DASH_L_c
**FBA:**	fdp_c → dhap_c + g3p_c
**DDPA:**	pep_c + e4p_c + h2o_c → 2dda7p_c + pi_c
**ICDHy:**	icit_c + nadp_c → akg_c + co2_c + nadph_c
**DHQTi:**	3dhq_c → 3dhsk_c + h2o_c
**DHQS:**	2dda7p_c → pi_c + 3dhq_c
**TKT2:**	g3p_c + f6p_c → xu5p_DASH_D_c + e4p_c
**HEX1:**	glc_DASH_D_c + atp_c → g6p_c + h_c + adp_c
**PGM:**	3pg_c → 2pg_c
**CSm:**	accoa_m + h2o_m + oaa_m → cit_m + h_m + coa_m
**PGI:**	g6p_c → f6p_c
**PHETA1:**	phpyr_c + glu_DASH_L_c → phe_DASH_L_c + akg_c
**OAAt2m:**	h_c + oaa_c → oaa_m + h_m
**PPNDH:**	pphn_c + h_c → phpyr_c + co2_c + h2o_c
**CITtbm:**	cit_m + pep_c → cit_c + pep_m
**SHKK:**	skm_c + atp_c → h_c + skm5p_c + adp_c
**GAPD:**	g3p_c + pi_c + nad_c → nadh_c + h_c + 13dpg_c
**PGK:**	13dpg_c + adp_c → 3pg_c + atp_c
**ACONT:**	cit_c → icit_c
**PYK:**	pep_c + h_c + adp_c → pyr_c + atp_c
**SHK3D:**	3dhsk_c + h_c + nadph_c → nadp_c + skm_c
**HCO3E:**	co2_c + h2o_c → hco3_c + h_c
**PC:**	hco3_c + pyr_c + atp_c → h_c + adp_c + pi_c + oaa_c
**CHORM:**	chor_c → pphn_c
**CHORS:**	3psme_c → chor_c + pi_c
**PFK:**	f6p_c + atp_c → fdp_c + h_c + adp_c

Pathway 2

**PSCVTi:**	pep_c + skm5p_c → pi_c + 3psme_c
**ENO:**	2pg_c → pep_c + h2o_c
**FBA:**	fdp_c → dhap_c + g3p_c
**DDPA:**	pep_c + e4p_c + h2o_c → 2dda7p_c + pi_c
**ICDHy:**	icit_c + nadp_c → akg_c + co2_c + nadph_c
**DHQTi:**	3dhq_c → 3dhsk_c + h2o_c
**DHQS:**	2dda7p_c → pi_c + 3dhq_c
**GLUK:**	glc_DASH_D_c + atp_c → h_c + g6p_DASH_B_c + adp_c
**TKT2:**	g3p_c + f6p_c → xu5p_DASH_D_c + e4p_c
**CHORS:**	3psme_c → chor_c + pi_c
**PGM:**	3pg_c → 2pg_c

**CSm:**	accoa_m + h2o_m + oaa_m → cit_m + h_m + coa_m
**PHETA1:**	phpyr_c + glu_DASH_L_c → phe_DASH_L_c + akg_c
**OAAt2m:**	h_c + oaa_c → oaa_m + h_m
**PPNDH:**	pphn_c + h_c → phpyr_c + co2_c + h2o_c
**CITtbm:**	cit_m + pep_c → cit_c + pep_m
**G6PI3:**	g6p_DASH_B_c → f6p_c
**PYK:**	pep_c + h_c + adp_c → pyr_c + atp_c
**GAPD:**	g3p_c + pi_c + nad_c → nadh_c + h_c + 13dpg_c
**PGK:**	13dpg_c + adp_c → 3pg_c + atp_c
**ACONT:**	cit_c → icit_c
**SHKK:**	skm_c + atp_c → h_c + skm5p_c + adp_c
**SHK3D:**	3dhsk_c + h_c + nadph_c → nadp_c + skm_c
**HCO3E:**	co2_c + h2o_c → hco3_c + h_c
**PC:**	hco3_c + pyr_c + atp_c → h_c + adp_c + pi_c + oaa_c
**CHORM:**	chor_c → pphn_c
**GLUDyi:**	akg_c + h_c + nadph_c + nh4_c → nadp_c + h2o_c + glu_DASH_L_c
**PFK:**	f6p_c + atp_c → fdp_c + h_c + adp_c

5.2 Pathways for catechol degradation

Below, we enlist the two pathways (shown in Figure 2 of main manuscript) found by MetQuest that degrade catechols in *Pseudomonas putida i*JN746. These two pathways represent the most diverse ways of catechol degradation. The reactions (in blue colour) represent the target reactions producing formate.

**Pathway 1**	
**3OADPCOAT**	succoa_c + 3oxoadp_c → succ_c + oxadpcoa_c
**3OXCOAT**	coa_c + oxadpcoa_c → succoa_c + accoa_c
**ACONT**	cit_c → icit_c
**AMPN**	h2o_c + amp_c → ade_c + r5p_c
**CATDOX**	o2_c + catechol_c → h_c + ccmuac_c
**catechol_e**	catechol_e → catechol_e
**CATECHOLtex**	catechol_e → catechol_p
**CATECHOLtpp**	catechol_p → catechol_c
**CS**	h2o_c + accoa_c + oaa_c → h_c + coa_c + cit_c
**ENO**	2pg_c → h2o_c + pep_c
**FUM**	mal__L_c → h2o_c + fum_c
**GAPD**	nad_c + pi_c + g3p_c → h_c + nadh_c + 13dpg_c
**ICL**	icit_c → succ_c + glx_c
**MALS**	h2o_c + accoa_c + glx_c → h_c + coa_c + mal__L_c
**MUCCYCI**	h_c + ccmuac_c → mucl_c
**MUCLI**	mucl_c → 5odhf2a_c
**O2tex**	o2_e → o2_p
**O2tpp**	o2_p → o2_c
**OXOAEL**	h2o_c + 5odhf2a_c → h_c + 3oxoadp_c
**PGK**	adp_c + 13dpg_c → atp_c + 3pg_c
**PGM**	3pg_c → 2pg_c
**PPC**	co2_c + h2o_c + pep_c → h_c + pi_c + oaa_c
**RPE**	ru5p__D_c → xu5p__D_c
**RPI**	r5p_c → ru5p__D_c
**TKT1**	r5p_c + xu5p__D_c → g3p_c + s7p_c
**Pathway 2**	
**4OD**	h_c + oxalc_c → co2_c + op4en_c
**4OT**	2hmc_c → oxalc_c
**ACALD**	coa_c + nad_c + acald_c → h_c + nadh_c + accoa_c
**ACONTa**	cit_c → h2o_c + acon_C_c
**ACONTb**	h2o_c + acon_C_c → icit_c
**ADA**	h_c + h2o_c + adn_c → nh4_c + ins_c
**ASPT**	asp__L_c → nh4_c + fum_c

**ASPTA**	glu__L_c + oaa_c → akg_c + asp__L_c
**CAT23DOX**	o2_c + catechol_c → 2hmcnsad_c
**catechol_e**	catechol_e → catechol_e
**CATECHOLtex**	catechol_e → catechol_p
**CATECHOLtpp**	catechol_p → catechol_c
**CS**	h2o_c + accoa_c + oaa_c → h_c + coa_c + cit_c
**GLUDy**	nadph_c + h_c + akg_c + nh4_c → nadp_c + h2o_c + glu__L_c
**H2CO3D**	co2_c + h2o_c → h2co3_c
**H2CO3D2**	h2co3_c → h_c + hco3_c
**HCO3E**	co2_c + h2o_c → h_c + hco3_c
**HMSD**	h2o_c + nad_c + 2hmcnsad_c → h_c + nadh_c + 2hmc_c
**HMSH**	h2o_c + 2hmcnsad_c → h_c + op4en_c + for_c
**HOPNTAL**	4h2opntn_c → acald_c + pyr_c
**ICDHyr**	nadp_c + icit_c → nadph_c + co2_c + akg_c
**O2tex**	o2_e → o2_p
**O2tpp**	o2_p → o2_c
**OP4ENH**	h2o_c + op4en_c → 4h2opntn_c
**PC**	atp_c + hco3_c + pyr_c → h_c + adp_c + pi_c + oaa_c

5.3 Comparison with other methods

The complete description of the reactions for the source–target pair listed in Table 1 (main manuscript) are given below:

1. **Source**: L-Arginine (C00062), **Target**: L- Citrulline (C00327)
Length: 2
R00551: C00062 + C00001 → C00086 + C00077
R00665: C00011 + C00014 + C00077 → C00327 + C00001

2. **Source**: Pyruvate (C00022), **Target**: Itaconate (C00490)
Length: 4
R02491: C01011 → C00531+ C00001
R00209: C00010+ C00003+ C00022 → C00024+ C00004+ C00011+ C00080
R00237: C01011 → C00024+ C00022
R02405: C00010+ C00490+ C00044 → C00531+ C00035+ C00009

3. **Source**: Pyruvate (C00022), **Target**: Itaconate (C00490)
Length: 5
R00351: C00010 + C00158 → C00036 + C00024 + C00001
R02243: C00417 → C00011 + C00490
R00209: C00010 + C00003 + C00022 → C00024 + C00004 + C00011 + C00080
R00217: C00036 → C00011 + C00022
R01325: C00158 → C00417 + C00001

4. **Source**: L-Tyrosine (C00082), **Target**: Naringenin (C00509)
Length: 5
R02446: C00509 → C06561
R00737: C00082 → C00811 + C00014
R01616: C00010 + C00811 + C00002 → C00013 + C00223 + C00020
R01613: C00223 + C00083 → C00010 + C00011 + C06561
R06641: C12441 + C00083 → C00010 + C00011

5. **Source**: L-Phenylalanine (C00079), **Target**: Resveratrol (C03582)
Length: 5
R02253: C00007 + C00423 + C00005 + C00080 → C00811 + C00001 + C00006
R00697: C00079 → C00423 + C00014
R01616: C00010 + C00811 + C00002 → C00013 + C00223 + C00020
R01614: C00083 + C00223 → C00010 + C03582 + C00011
R06641: C12441 + C00083 → C00010 + C00011

6. **Source**: Mevalonic acid (C00418), **Target**: Amorpha-4,11-diene (C16028)
Length: 7
R01658: C00129 + C00235 → C00341 + C00013
R03245: C00002 + C01107 → C01143 + C00008
R02245: C00418 + C00002 → C01107 + C00008
R01121: C00002 + C01143 → C00011 + C00008 + C00009 + C00129
R01123: C00129 → C00235
R07630: C00448 → C16028 + C00013
R02003: C00341 + C00129 → C00013 + C00448

5.4 Metabolic exchanges in a synthetic community of *E. coli* strains

In this section, we enlist the pathways containing metabolic exchanges between the two *E. coli* strains Δb2276 and Δb3708 (metabolite names respectively renamed). The reactions in blue coloured font represent the reactions pertaining to metabolic exchanges between the two strains.

Pathway 1
ACt2rpp: b3708 ac_p + b3708 h_p → b3708 ac_c + b3708 h_c
AMPN: b3708 amp_c + b3708 h2o_c → b3708 ade_c + b3708 r5p_c
AKGtex: b3708 akg_e → b3708 akg_p
ACt2rpp: b2276 ac_c + b2276 h_c → b2276 ac_p + b2276 h_p
ICDHyr: b2276 icit_c + b2276 nadp_c → b2276 akg_c + b2276 co2_c + b2276 nadph_c
ac_e: b2276 ac_e → ac_e
GLUDy: b3708 akg_c + b3708 h_c + b3708 nadph_c + b3708 nh4_c → b3708 glu_L_c + b3708 h2o_c + b3708 nadp_c
AKGt2rpp: b2276 akg_c + b2276 h_c → b2276 akg_p + b2276 h_p
ACtex: b2276 ac_p → b2276 ac_e
ACALD: b2276 accoa_c + b2276 h_c + b2276 nadh_c → b2276 acald_c + b2276 coa_c + b2276 nad_c
akg_e: akg_e → b3708 akg_e
ACONTa: b2276 cit_c → b2276 acon_C_c + b2276 h2o_c
ACS: b3708 ac_c + b3708 atp_c + b3708 coa_c → b3708 accoa_c + b3708 amp_c + b3708 ppi_c
ADD: b3708 ade_c + b3708 h2o_c + b3708 h_c → b3708 hxan_c + b3708 nh4_c
MALS: b2276 accoa_c + b2276 glx_c + b2276 h2o_c → b2276 coa_c + b2276 h_c + b2276 mal_L_c
MDH2: b2276 mal_L_c + b2276 q8_c → b2276 oaa_c + b2276 q8h2_c
ac_e: ac_e → b3708 ac_e
AKGt2rpp: b3708 akg_p + b3708 h_p → b3708 akg_c + b3708 h_c
akg_e: b2276 akg_e → akg_e
ACtex: b3708 ac_e → b3708 ac_p
AKGtex: b2276 akg_p → b2276 akg_e
ACONTb: b2276 acon_C_c + b2276 h2o_c → b2276 icit_c
ALDD2x: b2276 acald_c + b2276 h2o_c + b2276 nad_c → b2276 ac_c + b2276 h_c + b2276 nadh_c
CS: b2276 accoa_c + b2276 h2o_c + b2276 oaa_c → b2276 cit_c + b2276 coa_c + b2276 h_c
GLYCTO2: b2276 glyclt_c + b2276 q8_c → b2276 glx_c + b2276 q8h2_c

Pathway 2
ACt2rpp: b3708 ac_p + b3708 h_p → b3708 ac_c + b3708 h_c
AMPN: b3708 amp_c + b3708 h2o_c → b3708 ade_c + b3708 r5p_c
AKGtex: b3708 akg_e → b3708 akg_p
ACt2rpp: b2276 ac_c + b2276 h_c → b2276 ac_p + b2276 h_p
ICDHyr: b2276 icit_c + b2276 nadp_c → b2276 akg_c + b2276 co2_c + b2276 nadph_c
ac_e: b2276 ac_e → ac_e
GLUDy: b3708 akg_c + b3708 h_c + b3708 nadph_c + b3708 nh4_c → b3708 glu_L_c + b3708 h2o_c + b3708 nadp_c
AKGt2rpp: b2276 akg_c + b2276 h_c → b2276 akg_p + b2276 h_p
ACtex: b2276 ac_p → b2276 ac_e
ACALD: b2276 accoa_c + b2276 h_c + b2276 nadh_c → b2276 acald_c + b2276 coa_c + b2276 nad_c
akg_e: akg_e → b3708 akg_e
ACONTa: b2276 cit_c → b2276 acon_C_c + b2276 h2o_c
ACS: b3708 ac_c + b3708 atp_c + b3708 coa_c → b3708 accoa_c + b3708 amp_c + b3708 ppi_c

ALDD2y: b2276 acald_c + b2276 h2o_c + b2276 nadp_c → b2276 ac_c + b2276 h_c + b2276 nadph_c
ADD: b3708 ade_c + b3708 h2o_c + b3708 h_c → b3708 hxan_c + b3708 nh4_c
MALS: b2276 accoa_c + b2276 glx_c + b2276 h2o_c → b2276 coa_c + b2276 h_c + b2276 mal_L_c
MDH2: b2276 mal_L_c + b2276 q8_c → b2276 oaa_c + b2276 q8h2_c
ac_e: ac_e → b3708 ac_e
AKGt2rpp: b3708 akg_p + b3708 h_p → b3708 akg_c + b3708 h_c
akg_e: b2276 akg_e → akg_e
ACtex: b3708 ac_e → b3708 ac_p
AKGtex: b2276 akg_p → b2276 akg_e
ACONTb: b2276 acon_C_c + b2276 h2o_c → b2276 icit_c
CS: b2276 accoa_c + b2276 h2o_c + b2276 oaa_c → b2276 cit_c + b2276 coa_c + b2276 h_c
GLYCTO2: b2276 glyclt_c + b2276 q8_c → b2276 glx_c + b2276 q8h2_c

Pathway 3
ACt2rpp: b3708 ac_p + b3708 h_p → b3708 ac_c + b3708 h_c
AMPN: b3708 amp_c + b3708 h2o_c → b3708 ade_c + b3708 r5p_c
AKGtex: b3708 akg_e → b3708 akg_p
PTAr: b2276 accoa_c + b2276 pi_c → b2276 actp_c + b2276 coa_c
ACt2rpp: b2276 ac_c + b2276 h_c → b2276 ac_p + b2276 h_p
ICDHyr: b2276 icit_c + b2276 nadp_c → b2276 akg_c + b2276 co2_c + b2276 nadph_c
ac_e: b2276 ac_e → ac_e
GLUDy: b3708 akg_c + b3708 h_c + b3708 nadph_c + b3708 nh4_c → b3708 glu_L_c + b3708 h2o_c + b3708 nadp_c
AKGt2rpp: b2276 akg_c + b2276 h_c → b2276 akg_p + b2276 h_p
ACtex: b2276 ac_p → b2276 ac_e
akg_e: akg_e → b3708 akg_e
ACONTa: b2276 cit_c → b2276 acon_C_c + b2276 h2o_c
ACS: b3708 ac_c + b3708 atp_c + b3708 coa_c → b3708 accoa_c + b3708 amp_c + b3708 ppi_c
ADD: b3708 ade_c + b3708 h2o_c + b3708 h_c → b3708 hxan_c + b3708 nh4_c
MALS: b2276 accoa_c + b2276 glx_c + b2276 h2o_c → b2276 coa_c + b2276 h_c + b2276 mal_L_c
MDH2: b2276 mal_L_c + b2276 q8_c → b2276 oaa_c + b2276 q8h2_c
ACKr: b2276 actp_c + b2276 adp_c → b2276 ac_c + b2276 atp_c
ac_e: ac_e → b3708 ac_e
AKGt2rpp: b3708 akg_p + b3708 h_p → b3708 akg_c + b3708 h_c
akg_e: b2276 akg_e → akg_e
ACtex: b3708 ac_e → b3708 ac_p
AKGtex: b2276 akg_p → b2276 akg_e
ACONTb: b2276 acon_C_c + b2276 h2o_c → b2276 icit_c
CS: b2276 accoa_c + b2276 h2o_c + b2276 oaa_c → b2276 cit_c + b2276 coa_c + b2276 h_c
GLYCTO2: b2276 glyclt_c + b2276 q8_c → b2276 glx_c + b2276 q8h2_c

Pathway 4
ACt2rpp: b3708 ac_p + b3708 h_p → b3708 ac_c + b3708 h_c
ADA: b3708 adn_c + b3708 h2o_c + b3708 h_c → b3708 ins_c + b3708 nh4_c
NTD7: b3708 amp_c + b3708 h2o_c → b3708 adn_c + b3708 pi_c
AKGtex: b3708 akg_e → b3708 akg_p
ACt2rpp: b2276 ac_c + b2276 h_c → b2276 ac_p + b2276 h_p
ICDHyr: b2276 icit_c + b2276 nadp_c → b2276 akg_c + b2276 co2_c + b2276 nadph_c
ac_e: b2276 ac_e → ac_e
GLUDy: b3708 akg_c + b3708 h_c + b3708 nadph_c + b3708 nh4_c → b3708 glu_L_c + b3708 h2o_c + b3708 nadp_c
AKGt2rpp: b2276 akg_c + b2276 h_c → b2276 akg_p + b2276 h_p
ACtex: b2276 ac_p → b2276 ac_e
ACALD: b2276 accoa_c + b2276 h_c + b2276 nadh_c → b2276 acald_c + b2276 coa_c + b2276 nad_c
akg_e: akg_e → b3708 akg_e
ACONTa: b2276 cit_c → b2276 acon_C_c + b2276 h2o_c
ACS: b3708 ac_c + b3708 atp_c + b3708 coa_c → b3708 accoa_c + b3708 amp_c + b3708 ppi_c
MALS: b2276 accoa_c + b2276 glx_c + b2276 h2o_c → b2276 coa_c + b2276 h_c + b2276 mal_L_c

MDH2: b2276 mal_L_c + b2276 q8_c → b2276 oaa_c + b2276 q8h2_c
ac_e: ac_e → b3708 ac_e
AKGt2rpp: b3708 akg_p + b3708 h_p → b3708 akg_c + b3708 h_c
akg_e: b2276 akg_e → akg_e
ACtex: b3708 ac_e → b3708 ac_p
AKGtex: b2276 akg_p → b2276 akg_e
ACONTb: b2276 acon_C_c + b2276 h2o_c → b2276 icit_c
ALDD2x: b2276 acald_c + b2276 h2o_c + b2276 nad_c → b2276 ac_c + b2276 h_c + b2276 nadh_c
CS: b2276 accoa_c + b2276 h2o_c + b2276 oaa_c → b2276 cit_c + b2276 coa_c + b2276 h_c
GLYCTO2: b2276 glyclt_c + b2276 q8_c → b2276 glx_c + b2276 q8h2_c

Pathway 5
ACt2rpp: b3708 ac_p + b3708 h_p → b3708 ac_c + b3708 h_c
ADA: b3708 adn_c + b3708 h2o_c + b3708 h_c → b3708 ins_c + b3708 nh4_c
NTD7: b3708 amp_c + b3708 h2o_c → b3708 adn_c + b3708 pi_c
AKGtex: b3708 akg_e → b3708 akg_p
ACt2rpp: b2276 ac_c + b2276 h_c → b2276 ac_p + b2276 h_p
ICDHyr: b2276 icit_c + b2276 nadp_c → b2276 akg_c + b2276 co2_c + b2276 nadph_c
ac_e: b2276 ac_e → ac_e
GLUDy: b3708 akg_c + b3708 h_c + b3708 nadph_c + b3708 nh4_c → b3708 glu_L_c + b3708 h2o_c + b3708 nadp_c
AKGt2rpp: b2276 akg_c + b2276 h_c → b2276 akg_p + b2276 h_p
ACtex: b2276 ac_p → b2276 ac_e
ACALD: b2276 accoa_c + b2276 h_c + b2276 nadh_c → b2276 acald_c + b2276 coa_c + b2276 nad_c
akg_e: akg_e → b3708 akg_e
ACONTa: b2276 cit_c → b2276 acon_C_c + b2276 h2o_c
ACS: b3708 ac_c + b3708 atp_c + b3708 coa_c → b3708 accoa_c + b3708 amp_c + b3708 ppi_c
ALDD2y: b2276 acald_c + b2276 h2o_c + b2276 nadp_c → b2276 ac_c + b2276 h_c + b2276 nadph_c
MALS: b2276 accoa_c + b2276 glx_c + b2276 h2o_c → b2276 coa_c + b2276 h_c + b2276 mal_L_c
MDH2: b2276 mal_L_c + b2276 q8_c → b2276 oaa_c + b2276 q8h2_c
ac_e: ac_e → b3708 ac_e
AKGt2rpp: b3708 akg_p + b3708 h_p → b3708 akg_c + b3708 h_c
akg_e: b2276 akg_e → akg_e
ACtex: b3708 ac_e → b3708 ac_p
AKGtex: b2276 akg_p → b2276 akg_e
ACONTb: b2276 acon_C_c + b2276 h2o_c → b2276 icit_c
CS: b2276 accoa_c + b2276 h2o_c + b2276 oaa_c → b2276 cit_c + b2276 coa_c + b2276 h_c
GLYCTO2: b2276 glyclt_c + b2276 q8_c → b2276 glx_c + b2276 q8h2_c

Pathway 6
ACt2rpp: b3708 ac_p + b3708 h_p → b3708 ac_c + b3708 h_c
ADA: b3708 adn_c + b3708 h2o_c + b3708 h_c → b3708 ins_c + b3708 nh4_c
NTD7: b3708 amp_c + b3708 h2o_c → b3708 adn_c + b3708 pi_c
AKGtex: b3708 akg_e → b3708 akg_p
PTAr: b2276 accoa_c + b2276 pi_c → b2276 actp_c + b2276 coa_c
ACt2rpp: b2276 ac_c + b2276 h_c → b2276 ac_p + b2276 h_p
ICDHyr: b2276 icit_c + b2276 nadp_c → b2276 akg_c + b2276 co2_c + b2276 nadph_c
ac_e: b2276 ac_e → ac_e
GLUDy: b3708 akg_c + b3708 h_c + b3708 nadph_c + b3708 nh4_c → b3708 glu_L_c + b3708 h2o_c + b3708 nadp_c
AKGt2rpp: b2276 akg_c + b2276 h_c → b2276 akg_p + b2276 h_p
ACtex: b2276 ac_p → b2276 ac_e
akg_e: akg_e → b3708 akg_e
ACONTa: b2276 cit_c → b2276 acon_C_c + b2276 h2o_c
ACS: b3708 ac_c + b3708 atp_c + b3708 coa_c → b3708 accoa_c + b3708 amp_c + b3708 ppi_c
MALS: b2276 accoa_c + b2276 glx_c + b2276 h2o_c → b2276 coa_c + b2276 h_c + b2276 mal_L_c
MDH2: b2276 mal_L_c + b2276 q8_c → b2276 oaa_c + b2276 q8h2_c
ACKr: b2276 actp_c + b2276 adp_c → b2276 ac_c + b2276 atp_c

ac_e: ac_e → b3708 ac_e
AKGt2rpp: b3708 akg_p + b3708 h_p → b3708 akg_c + b3708 h_c
akg_e: b2276 akg_e → akg_e
ACtex: b3708 ac_e → b3708 ac_p
AKGtex: b2276 akg_p → b2276 akg_e
ACONTb: b2276 acon_C_c + b2276 h2o_c → b2276 icit_c
CS: b2276 accoa_c + b2276 h2o_c + b2276 oaa_c → b2276 cit_c + b2276 coa_c + b2276 h_c
GLYCTO2: b2276 glyclt_c + b2276 q8_c → b2276 glx_c + b2276 q8h2_c

Pathway 7
ACt2rpp: b3708 ac_p + b3708 h_p → b3708 ac_c + b3708 h_c
AMPN: b3708 amp_c + b3708 h2o_c → b3708 ade_c + b3708 r5p_c
AKGtex: b3708 akg_e → b3708 akg_p
ACt2rpp: b2276 ac_c + b2276 h_c → b2276 ac_p + b2276 h_p
ICDHyr: b2276 icit_c + b2276 nadp_c → b2276 akg_c + b2276 co2_c + b2276 nadph_c
ac_e: b2276 ac_e → ac_e
GLUDy: b3708 akg_c + b3708 h_c + b3708 nadph_c + b3708 nh4_c → b3708 glu_L_c + b3708 h2o_c + b3708 nadp_c
MDH: b2276 mal_L_c + b2276 nad_c → b2276 h_c + b2276 nadh_c + b2276 oaa_c
AKGt2rpp: b2276 akg_c + b2276 h_c → b2276 akg_p + b2276 h_p
ACtex: b2276 ac_p → b2276 ac_e
ACALD: b2276 accoa_c + b2276 h_c + b2276 nadh_c → b2276 acald_c + b2276 coa_c + b2276 nad_c
akg_e: akg_e → b3708 akg_e
ACONTa: b2276 cit_c → b2276 acon_C_c + b2276 h2o_c
ACS: b3708 ac_c + b3708 atp_c + b3708 coa_c → b3708 accoa_c + b3708 amp_c + b3708 ppi_c
ADD: b3708 ade_c + b3708 h2o_c + b3708 h_c → b3708 hxan_c + b3708 nh4_c
MALS: b2276 accoa_c + b2276 glx_c + b2276 h2o_c → b2276 coa_c + b2276 h_c + b2276 mal_L_c
ac_e: ac_e → b3708 ac_e
AKGt2rpp: b3708 akg_p + b3708 h_p → b3708 akg_c + b3708 h_c
akg_e: b2276 akg_e → akg_e
ACtex: b3708 ac_e → b3708 ac_p
AKGtex: b2276 akg_p → b2276 akg_e
ACONTb: b2276 acon_C_c + b2276 h2o_c → b2276 icit_c
ALDD2x: b2276 acald_c + b2276 h2o_c + b2276 nad_c → b2276 ac_c + b2276 h_c + b2276 nadh_c
CS: b2276 accoa_c + b2276 h2o_c + b2276 oaa_c → b2276 cit_c + b2276 coa_c + b2276 h_c
GLYCTO2: b2276 glyclt_c + b2276 q8_c → b2276 glx_c + b2276 q8h2_c

Pathway 8
ACt2rpp: b3708 ac_p + b3708 h_p → b3708 ac_c + b3708 h_c
AMPN: b3708 amp_c + b3708 h2o_c → b3708 ade_c + b3708 r5p_c
AKGtex: b3708 akg_e → b3708 akg_p
ACt2rpp: b2276 ac_c + b2276 h_c → b2276 ac_p + b2276 h_p
ICDHyr: b2276 icit_c + b2276 nadp_c → b2276 akg_c + b2276 co2_c + b2276 nadph_c
ac_e: b2276 ac_e → ac_e
GLUDy: b3708 akg_c + b3708 h_c + b3708 nadph_c + b3708 nh4_c → b3708 glu_L_c + b3708 h2o_c + b3708 nadp_c
MDH: b2276 mal_L_c + b2276 nad_c → b2276 h_c + b2276 nadh_c + b2276 oaa_c
AKGt2rpp: b2276 akg_c + b2276 h_c → b2276 akg_p + b2276 h_p
ACtex: b2276 ac_p → b2276 ac_e
ACALD: b2276 accoa_c + b2276 h_c + b2276 nadh_c → b2276 acald_c + b2276 coa_c + b2276 nad_c
akg_e: akg_e → b3708 akg_e
ACONTa: b2276 cit_c → b2276 acon_C_c + b2276 h2o_c
ACS: b3708 ac_c + b3708 atp_c + b3708 coa_c → b3708 accoa_c + b3708 amp_c + b3708 ppi_c
ALDD2y: b2276 acald_c + b2276 h2o_c + b2276 nadp_c → b2276 ac_c + b2276 h_c + b2276 nadph_c
ADD: b3708 ade_c + b3708 h2o_c + b3708 h_c → b3708 hxan_c + b3708 nh4_c
MALS: b2276 accoa_c + b2276 glx_c + b2276 h2o_c → b2276 coa_c + b2276 h_c + b2276 mal_L_c
ac_e: ac_e → b3708 ac_e
AKGt2rpp: b3708 akg_p + b3708 h_p → b3708 akg_c + b3708 h_c

akg_e: b2276 akg_e → akg_e
ACtex: b3708 ac_e → b3708 ac_p
AKGtex: b2276 akg_p → b2276 akg_e
ACONTb: b2276 acon_C_c + b2276 h2o_c → b2276 icit_c
CS: b2276 accoa_c + b2276 h2o_c + b2276 oaa_c → b2276 cit_c + b2276 coa_c + b2276 h_c
GLYCTO2: b2276 glyclt_c + b2276 q8_c → b2276 glx_c + b2276 q8h2_c

Pathway 9
ACt2rpp: b3708 ac_p + b3708 h_p → b3708 ac_c + b3708 h_c
AMPN: b3708 amp_c + b3708 h2o_c → b3708 ade_c + b3708 r5p_c
AKGtex: b3708 akg_e → b3708 akg_p
PTAr: b2276 accoa_c + b2276 pi_c → b2276 actp_c + b2276 coa_c
ACt2rpp: b2276 ac_c + b2276 h_c → b2276 ac_p + b2276 h_p
ICDHyr: b2276 icit_c + b2276 nadp_c → b2276 akg_c + b2276 co2_c + b2276 nadph_c
ac_e: b2276 ac_e → ac_e
GLUDy: b3708 akg_c + b3708 h_c + b3708 nadph_c + b3708 nh4_c → b3708 glu_L_c + b3708 h2o_c + b3708 nadp_c
MDH: b2276 mal_L_c + b2276 nad_c → b2276 h_c + b2276 nadh_c + b2276 oaa_c
AKGt2rpp: b2276 akg_c + b2276 h_c → b2276 akg_p + b2276 h_p
ACtex: b2276 ac_p → b2276 ac_e
akg_e: akg_e → b3708 akg_e
ACONTa: b2276 cit_c → b2276 acon_C_c + b2276 h2o_c
ACS: b3708 ac_c + b3708 atp_c + b3708 coa_c → b3708 accoa_c + b3708 amp_c + b3708 ppi_c
ADD: b3708 ade_c + b3708 h2o_c + b3708 h_c → b3708 hxan_c + b3708 nh4_c
MALS: b2276 accoa_c + b2276 glx_c + b2276 h2o_c → b2276 coa_c + b2276 h_c + b2276 mal_L_c
ACKr: b2276 actp_c + b2276 adp_c → b2276 ac_c + b2276 atp_c
ac_e: ac_e → b3708 ac_e
AKGt2rpp: b3708 akg_p + b3708 h_p → b3708 akg_c + b3708 h_c
akg_e: b2276 akg_e → akg_e
ACtex: b3708 ac_e → b3708 ac_p
AKGtex: b2276 akg_p → b2276 akg_e
ACONTb: b2276 acon_C_c + b2276 h2o_c → b2276 icit_c
CS: b2276 accoa_c + b2276 h2o_c + b2276 oaa_c → b2276 cit_c + b2276 coa_c + b2276 h_c
GLYCTO2: b2276 glyclt_c + b2276 q8_c → b2276 glx_c + b2276 q8h2_c

Pathway 10
ACt2rpp: b3708 ac_p + b3708 h_p → b3708 ac_c + b3708 h_c
ADA: b3708 adn_c + b3708 h2o_c + b3708 h_c → b3708 ins_c + b3708 nh4_c
NTD7: b3708 amp_c + b3708 h2o_c → b3708 adn_c + b3708 pi_c
AKGtex: b3708 akg_e → b3708 akg_p
ACt2rpp: b2276 ac_c + b2276 h_c → b2276 ac_p + b2276 h_p
ICDHyr: b2276 icit_c + b2276 nadp_c → b2276 akg_c + b2276 co2_c + b2276 nadph_c
ac_e: b2276 ac_e → ac_e
GLUDy: b3708 akg_c + b3708 h_c + b3708 nadph_c + b3708 nh4_c → b3708 glu_L_c + b3708 h2o_c + b3708 nadp_c
MDH: b2276 mal_L_c + b2276 nad_c → b2276 h_c + b2276 nadh_c + b2276 oaa_c
AKGt2rpp: b2276 akg_c + b2276 h_c → b2276 akg_p + b2276 h_p
ACtex: b2276 ac_p → b2276 ac_e
ACALD: b2276 accoa_c + b2276 h_c + b2276 nadh_c → b2276 acald_c + b2276 coa_c + b2276 nad_c
akg_e: akg_e → b3708 akg_e
ACONTa: b2276 cit_c → b2276 acon_C_c + b2276 h2o_c
ACS: b3708 ac_c + b3708 atp_c + b3708 coa_c → b3708 accoa_c + b3708 amp_c + b3708 ppi_c
MALS: b2276 accoa_c + b2276 glx_c + b2276 h2o_c → b2276 coa_c + b2276 h_c + b2276 mal_L_c
ac_e: ac_e → b3708 ac_e
AKGt2rpp: b3708 akg_p + b3708 h_p → b3708 akg_c + b3708 h_c
akg_e: b2276 akg_e → akg_e
ACtex: b3708 ac_e → b3708 ac_p
AKGtex: b2276 akg_p → b2276 akg_e

ACONTb: b2276 acon_C_c + b2276 h2o_c → b2276 icit_c

ALDD2x: b2276 acald_c + b2276 h2o_c + b2276 nad_c → b2276 ac_c + b2276 h_c + b2276 nadh_c

CS: b2276 accoa_c + b2276 h2o_c + b2276 oaa_c → b2276 cit_c + b2276 coa_c + b2276 h_c

GLYCTO2: b2276 glyclt_c + b2276 q8_c → b2276 glx_c + b2276 q8h2_c

Pathway 11

ACt2rpp: b3708 ac_p + b3708 h_p → b3708 ac_c + b3708 h_c

ADA: b3708 adn_c + b3708 h2o_c + b3708 h_c → b3708 ins_c + b3708 nh4_c

NTD7: b3708 amp_c + b3708 h2o_c → b3708 adn_c + b3708 pi_c

AKGtex: b3708 akg_e → b3708 akg_p

ACt2rpp: b2276 ac_c + b2276 h_c → b2276 ac_p + b2276 h_p

ICDHyr: b2276 icit_c + b2276 nadp_c → b2276 akg_c + b2276 co2_c + b2276 nadph_c

ac_e: b2276 ac_e → ac_e

GLUDy: b3708 akg_c + b3708 h_c + b3708 nadph_c + b3708 nh4_c → b3708 glu_L_c + b3708 h2o_c + b3708 nadp_c

MDH: b2276 mal_L_c + b2276 nad_c → b2276 h_c + b2276 nadh_c + b2276 oaa_c

AKGt2rpp: b2276 akg_c + b2276 h_c → b2276 akg_p + b2276 h_p

ACtex: b2276 ac_p → b2276 ac_e

ACALD: b2276 accoa_c + b2276 h_c + b2276 nadh_c → b2276 acald_c + b2276 coa_c + b2276 nad_c

akg_e: akg_e → b3708 akg_e

ACONTa: b2276 cit_c → b2276 acon_C_c + b2276 h2o_c

ACS: b3708 ac_c + b3708 atp_c + b3708 coa_c → b3708 accoa_c + b3708 amp_c + b3708 ppi_c

ALDD2y: b2276 acald_c + b2276 h2o_c + b2276 nadp_c → b2276 ac_c + b2276 h_c + b2276 nadph_c

MALS: b2276 accoa_c + b2276 glx_c + b2276 h2o_c → b2276 coa_c + b2276 h_c + b2276 mal_L_c

ac_e: ac_e → b3708 ac_e

AKGt2rpp: b3708 akg_p + b3708 h_p → b3708 akg_c + b3708 h_c

akg_e: b2276 akg_e → akg_e

ACtex: b3708 ac_e → b3708 ac_p

AKGtex: b2276 akg_p → b2276 akg_e

ACONTb: b2276 acon_C_c + b2276 h2o_c → b2276 icit_c

CS: b2276 accoa_c + b2276 h2o_c + b2276 oaa_c → b2276 cit_c + b2276 coa_c + b2276 h_c

GLYCTO2: b2276 glyclt_c + b2276 q8_c → b2276 glx_c + b2276 q8h2_c

Pathway 12

ACt2rpp: b3708 ac_p + b3708 h_p → b3708 ac_c + b3708 h_c

ADA: b3708 adn_c + b3708 h2o_c + b3708 h_c → b3708 ins_c + b3708 nh4_c

NTD7: b3708 amp_c + b3708 h2o_c → b3708 adn_c + b3708 pi_c

AKGtex: b3708 akg_e → b3708 akg_p

PTAr: b2276 accoa_c + b2276 pi_c → b2276 actp_c + b2276 coa_c

ACt2rpp: b2276 ac_c + b2276 h_c → b2276 ac_p + b2276 h_p

ICDHyr: b2276 icit_c + b2276 nadp_c → b2276 akg_c + b2276 co2_c + b2276 nadph_c

ac_e: b2276 ac_e → ac_e

GLUDy: b3708 akg_c + b3708 h_c + b3708 nadph_c + b3708 nh4_c → b3708 glu_L_c + b3708 h2o_c + b3708 nadp_c

MDH: b2276 mal_L_c + b2276 nad_c → b2276 h_c + b2276 nadh_c + b2276 oaa_c

AKGt2rpp: b2276 akg_c + b2276 h_c → b2276 akg_p + b2276 h_p

ACtex: b2276 ac_p → b2276 ac_e

akg_e: akg_e → b3708 akg_e

ACONTa: b2276 cit_c → b2276 acon_C_c + b2276 h2o_c

ACS: b3708 ac_c + b3708 atp_c + b3708 coa_c → b3708 accoa_c + b3708 amp_c + b3708 ppi_c

MALS: b2276 accoa_c + b2276 glx_c + b2276 h2o_c → b2276 coa_c + b2276 h_c + b2276 mal_L_c

ACKr: b2276 actp_c + b2276 adp_c → b2276 ac_c + b2276 atp_c

ac_e: ac_e → b3708 ac_e

AKGt2rpp: b3708 akg_p + b3708 h_p → b3708 akg_c + b3708 h_c

akg_e: b2276 akg_e → akg_e

ACtex: b3708 ac_e → b3708 ac_p

AKGtex: b2276 akg_p → b2276 akg_e

ACONTb: b2276 acon_C_c + b2276 h2o_c → b2276 icit_c

CS: b2276 accoa_c + b2276 h2o_c + b2276 oaa_c → b2276 cit_c + b2276 coa_c + b2276 h_c
GLYCTO2: b2276 glyclt_c + b2276 q8_c → b2276 glx_c + b2276 q8h2_c

Supplementary References

[1] Šīrava, M. *et al.* BioMiner–modeling, analyzing, and visualizing biochemical pathways and networks. *Bioinformatics* **18** **Suppl 2**, S219–S230 (2002).

[2] McShan, D. C., Rao, S. & Shah, I. PathMiner: Predicting metabolic pathways by heuristic search. *Bioinformatics* **19**, 1692–1698 (2003).

[3] Handorf, T. & Ebenhöh, O. MetaPath Online: A web server implementation of the network expansion algorithm. *Nucleic Acids Res.* **35**, 613–618 (2007).

[4] Brohee, S. *et al.* NeAT: a toolbox for the analysis of biological networks, clusters, classes and pathways. *Nucleic Acids Res.* **36**, W444–W451 (2008).

[5] Mithani, A., Preston, G. M. & Hein, J. Rahnuma: Hypergraph-based tool for metabolic pathway prediction and network comparison. *Bioinformatics* **25**, 1831–1832 (2009).

[6] Pitkänen, E., Jouhten, P. & Rousu, J. Inferring branching pathways in genome-scale metabolic networks. *BMC Syst. Biol.* **3**, 103 (2009).

[7] Chou, C. H., Chang, W. C., Chiu, C. M., Huang, C. C. & Huang, H. D. FMM: A web server for metabolic pathway reconstruction and comparative analysis. *Nucleic Acids Res.* **37**, 129–134 (2009).

[8] Moriya, Y. *et al.* PathPred: An enzyme-catalyzed metabolic pathway prediction server. *Nucleic Acids Res.* **38**, 138–143 (2010).

[9] Heath, A. P., Bennett, G. N. & Kavraki, L. E. An Algorithm for Efficient Identification of Branched Metabolic Pathways. *J. Comput. Biol.* **18**, 1575–1597 (2011).

[10] Xia, D. *et al.* MRSD: A web server for Metabolic Route Search and Design. *Bioinformatics* **27**, 1581–1582 (2011).

[11] McClymont, K. & Soyer, O. S. Metabolic tinker: an online tool for guiding the design of synthetic metabolic pathways. *Nucleic Acids Res.* **41**, e113 (2013).

[12] Latendresse, M., Krummenacker, M. & Karp, P. D. Optimal metabolic route search based on atom mappings. *Bioinformatics* **30**, 2043–2050 (2014).

[13] Vieira, G., Carnicer, M., Portais, J.-C. & Heux, S. FindPath: a Matlab solution for in silico design of synthetic metabolic pathways. *Bioinformatics* **30**, 2986–2988 (2014).

[14] Hadadi, N., Hafner, J., Shajkofci, A., Zisaki, A. & Hatzimanikatis, V. ATLAS of Biochemistry: A Repository of All Possible Biochemical Reactions for Synthetic Biology and Metabolic Engineering Studies. *ACS Synth. Biol.* **5**, 1155–1166 (2016).

[15] Kuwahara, H., Alazmi, M., Cui, X. & Gao, X. MRE: a web tool to suggest foreign enzymes for the biosynthesis pathway design with competing endogenous reactions in mind. *Nucleic Acids Res.* **44**, W217–W225 (2016).

[16] Ebrahim, A., Lerman, J. a., Palsson, B. O. & Hyduke, D. R. COBRApy: COnstraints-Based Reconstruction and Analysis for Python. *BMC Syst. Biol.* **7**, 74 (2013).

[17] Hagberg, A. A., Schult, D. A. & Swart, P. J. Exploring Network Structure, Dynamics, and Function using NetworkX. In Varoquaux, G., Vaught, T. & Millman, J. (eds.) *Proc. 7th Python Sci. Conf.*, 11–15 (Pasadena, CA USA, 2008).
